# Grain-boundary segregation of magnesium in doped cuprous oxide and impact on electrical transport properties

**DOI:** 10.1038/s41598-021-86969-7

**Published:** 2021-04-08

**Authors:** João Resende, Van-Son Nguyen, Claudia Fleischmann, Lorenzo Bottiglieri, Stéphane Brochen, Wilfried Vandervorst, Wilfried Favre, Carmen Jiménez, Jean-Luc Deschanvres, Ngoc Duy Nguyen

**Affiliations:** 1 Univ. Grenoble Alpes, CNRS, Grenoble INP, LMGP, 38000 Grenoble, France; 2 Département de Physique, CESAM/Q-MAT, SPIN, Université de Liège, 4000 Liège, Belgium; 3 CEA, INES, LITEN, 50 Avenue du lac Léman, 73375 Le Bourget-du-lac, France; 4 IMEC, Kapeldreef 75, 3001 Heverlee, Belgium; 5 Instituut Voor Kern- en Stralingsfysica, KU Leuven, Celestijnenlaan 200D, 3001 Leuven, Belgium

**Keywords:** Materials for devices, Materials for energy and catalysis, Techniques and instrumentation

## Abstract

In this study, we report the segregation of magnesium in the grain boundaries of magnesium-doped cuprous oxide (Cu_2_O:Mg) thin films as revealed by atom probe tomography and the consequences of the dopant presence on the temperature-dependent Hall effect properties. The incorporation of magnesium as a divalent cation was achieved by aerosol-assisted metal organic chemical vapour deposition, followed by thermal treatments under oxidizing conditions. We observe that, in comparison with intrinsic cuprous oxide, the electronic transport is improved in Cu_2_O:Mg with a reduction of resistivity to 13.3 ± 0.1 Ω cm, despite the reduction of hole mobility in the doped films, due to higher grain-boundary scattering. The Hall carrier concentration dependence with temperature showed the presence of an acceptor level associated with an ionization energy of 125 ± 9 meV, similar to the energy value of a large size impurity−vacancy complex. Atom probe tomography shows a magnesium incorporation of 5%, which is substantially present at the grain boundaries of the Cu_2_O.

## Introduction

Cuprous oxide (Cu_2_O) thin films have attracted attention for photovoltaic and transparent electronic applications due to their tuneable direct bandgap, p-type conductivity, non-toxicity, abundance and low-cost productivity^1,2^. The p-type behavior of cuprous oxide arises from the special configuration of the valence band, formed by 3d^10^ levels of the Cu^+^ cation. The existence of these different levels contribute to a less localized population of holes, which improves the mobility of these charges^3^. For the generation of holes, the copper vacancies, $V_{Cu}^{^{\prime}}$, are considered as the most favorable defect from the point of view of formation energy. They are generally created during post-deposition annealing treatments under oxidizing conditions^4–6^. At temperatures below 300 °C, Cu_2_O thin films show a decrease of the resistivity down to values as low as 100 Ω cm, due to an increase of the p-type charge carrier concentration which can reach up to 10^16^ cm^−3^^4,7^. To further increase the concentration of charge carriers, copper oxide can be doped with several elements such as nitrogen^8–11^, sodium^12^, strontium^13^, or magnesium^14–16^, which also impact their optical performances compared to pure Cu_2_O. The use of these dopants seems to reduce the resistivity of Cu_2_O thin films, by increasing the density of holes from 10^17^ to 10^19^ cm^−3^. Additionally, they are responsible for the creation of an extra acceptor level in Cu_2_O^9,13^. In the specific case of magnesium incorporation in Cu_2_O, this dopant showed a strong effect on the resistivity, reaching values as low as 7 Ω cm^16^, by increasing the charge carrier density up to 8.1 × 10^17^ cm^−3^. Besides, this dopant increases the stability of the Cu_2_O phase under thermal oxidizing treatments^17^. Additionally, several all-oxide solar cells have been produced with Mg-doped Cu_2_O, by using TiO_2_^14^ and ZnO^15^ as n-type counterparts, reporting efficiency values close to 1%. Nevertheless, there lacks a detailed understanding of the dopant location in the structure of Cu_2_O and of the underlying mechanism at the origin of the increase of the carrier density.

In this study, we analyse the spatial distribution of magnesium as dopant in polycrystalline copper oxide by atom probe tomography and its direct impact on the electrical transport properties by temperature-dependent Hall effect. Additionally, intrinsic and magnesium-doped Cu_2_O (Cu_2_O:Mg) thin films have been thermally annealed in air at temperatures above 250 °C to further investigate the generation of charge carriers in the thin films.

## Results

The as-deposited Cu_2_O and Cu_2_O:Mg thin films were routinely characterized in order to measure the thickness by scanning electron microscope (SEM) cross section imaging and the resistivity by the Van der Pauw method. The intrinsic Cu_2_O thin films presented a thickness of 240 ± 10 nm with a resistivity of 93 ± 1 Ω cm, while the Cu_2_O:Mg films were 180 ± 10 nm thick, with a resistivity of 61.3 ± 0.4 Ω cm.

### Magnesium location in copper oxide thin films

**Figure 1:**
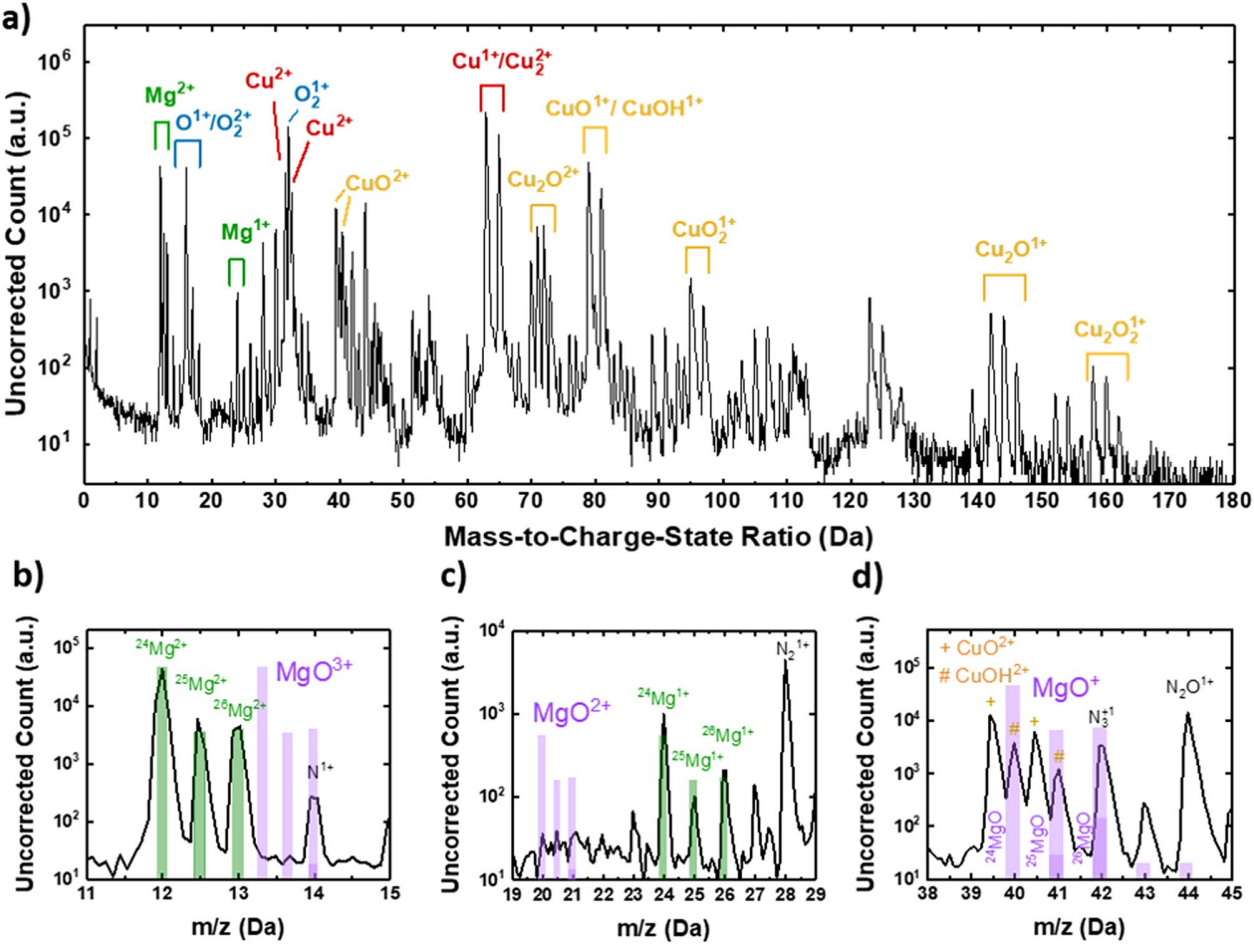
Atom probe mass spectra averaged over the volume corresponding to the as-deposited Cu_2_O:Mg film. The ions collected from the Si substrate are not shown for clarity: (**a**) full mass spectrum with the relevant atomic and molecular ion species highlighted; (**b**–**d**) show selected regions of the spectrum shown in (**a**), in which the location and abundance of the peaks originating from Mg^nx^ ions and MgO_x_^nx^ ions shown by green and purple bars, respectively.

Atom probe tomography (APT) is ideally suited to study the 3-dimensional dopant distribution^18–23^. The basic principle of APT, including the procedure for building the tomographic reconstruction, is described in detail elsewhere^24–26^. APT analysis of the as-deposited Cu_2_O:Mg film yielded an average Mg incorporation of 5%, computed with respect to both cations (Mg/(Mg + Cu)). Both singly Mg^+^ and doubly charged Mg^2+^ ions are observed in the APT mass spectrum (Fig. 1a), with a higher intensity of the doubly charged ions. No traces of magnesium oxides were found in the mass spectrum, as depicted in Fig. 1b–d by purple bars, which represent the location and expected peak ratio based on the natural isotopic abundance of the MgO^+^, MgO^2+^ and MgO^3+^ ions. The peaks at 40 and 41 Da (Fig. 1d, represented by #) were assigned to CuOH^2+^ due to their matching ratio with the isotopic abundance of the Cu hydride rather than the MgO^+^ ion^25,26^. No carbon contamination from the AA-MOCVD process was detected in the APT mass spectrum. The peaks at 12 Da (=C^+^ or Mg^2+^), 12.5 Da and 13 Da show a very good agreement with the expected isotopic ratio of Mg^2+^, ruling out a contribution of C^+^ ions to the peak at 12 Da.

The mass spectrum further contains various singly (n = 1) and doubly (n = 2) charged Cu molecular ions such as CuO^n+^, Cu_2_O^n+^, CuO_2_^n+^, Cu_2_O_2_^n+^ and CuOHn^+^. Charged molecules, including hydride ions, are commonly detected when field-evaporating covalently or ionically bonded materials^27,28^, and hence their presence in the mass spectrum does not reflect the chemistry of the Cu_2_O film. Indeed, previous studies on Cu_2_O:Mg thin films confirm the sole presence of crystalline cuprous oxide, Cu_2_O, by XRD, Raman, TEM and FTIR^14,16,17^. Considering the complex processes that are intrinsically related to field evaporation and/or the behaviour of molecular ions in high electric fields (e.g. field-induced dissociation^27^), it is typically very cumbersome to draw reliable and quantitative information on the charge state of the analysed material based on APT mass spectra. Other unidentified peaks in Fig. 1a correspond to combinations of complex ions, as no match was possible to be established.

**Figure 2:**
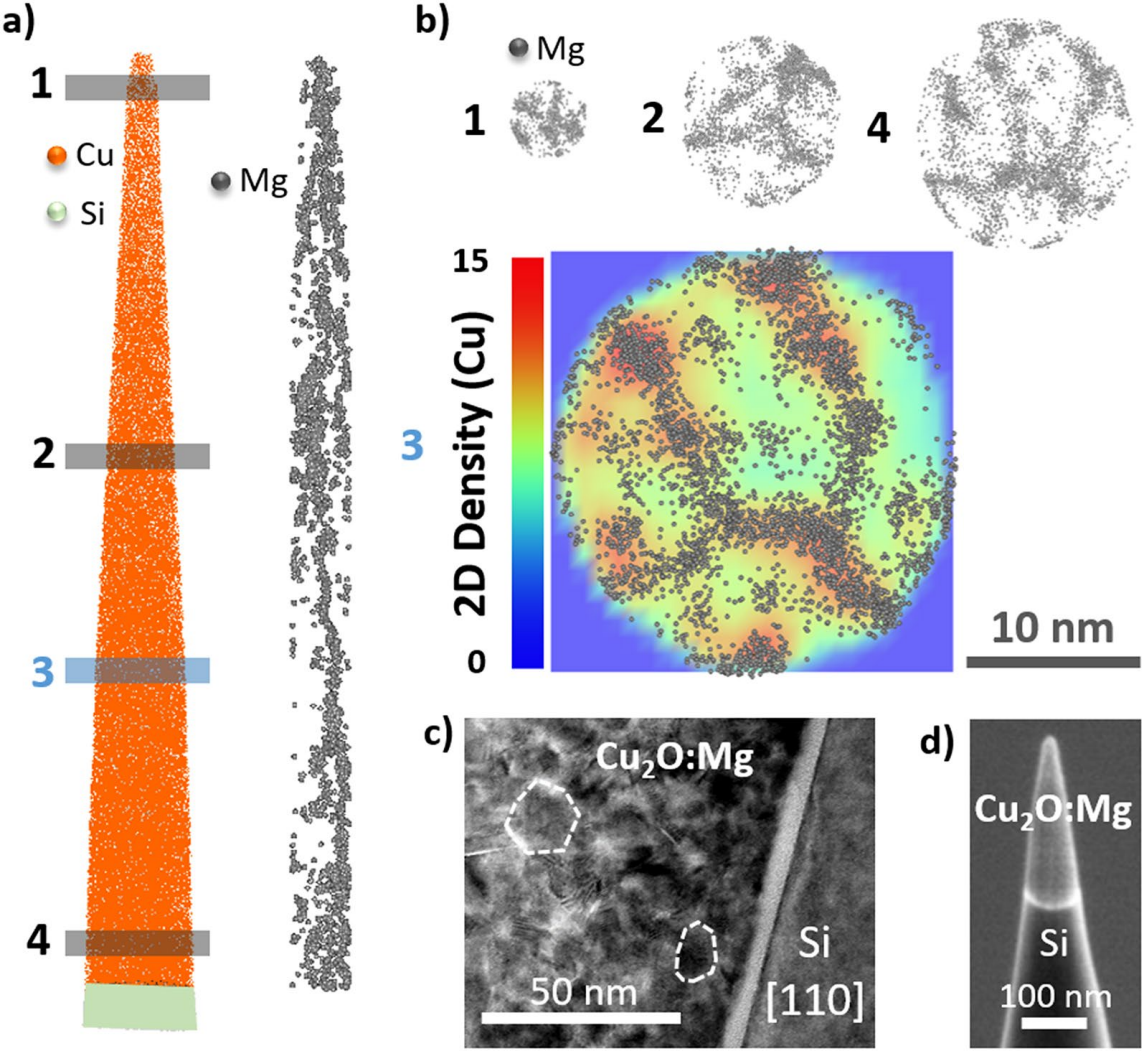
3D atomic map of the as-deposited Cu_2_O:Mg film for which the tip shape shown in (**d**) was used to guide the tomographic reconstruction. (**a**) 3D atomic map of Cu and Si atoms is presented by orange and light green dots, respectively (left-hand side) and distribution of Mg in a 1 nm thick slice parallel to the APT specimen axis is shown, presenting a cross-sectional view of the film in dark grey dots (right-hand side). (**b**) 10 nm thick slices taken at different positions from top to bottom (number 1–4) represent a planar view of the film. In 1, 2 and 4 the 2D Mg ion maps are shown and in 3 this is superimposed with the Cu ion 2D density color map. (**c**) TEM micrograph of Cu_2_O:Mg thin film cross-section deposited on silicon and (**d**) SEM micrograph of the Cu_2_O:Mg specimen before APT analysis.

The spatial distribution of the Mg atoms is inhomogeneous throughout the Cu_2_O film, visible in Fig. 2a, with a local enrichment that resembles the grain boundary (GB) network of the film, with a grain size between 10 and 20 nm, as presented in Fig. 2c of TEM imaging. The grains present a round-like shape, with some straight edges, contrarily to the common columnar growth of intrinsic Cu_2_O oriented perpendicular to the substrate prepared by MOCVD^29^.

Representative 2-dimensional (2D) Mg ion maps are shown in both cross-section and planar views of the film in Fig. 2a,b, respectively, as well as the tip SEM image before APT analysis, visible in Fig. 2d. The planar Mg distribution correlates well with regions of higher atomic density in the 2D Cu ion density map, Fig. 2b slice 3, suggesting an Mg segregation into grain boundaries. Atomic density variations in the 3D volume reconstruction (or on the detector) can be ascribed to ion trajectory aberrations (deflection of the ions) and associated local magnification effects^20,25,30–32^. These typically originate from local disturbances in the electric field distribution, which often result from dissimilarities in the local curvature of the APT tip developed around microstructural features such as grain boundaries, interfaces, precipitates etc.

The local presence of Mg in the edges of the grains can be observed in Fig. 2b slice 3 and 4, where the dopant shows the shape of vertical grain boundaries to the axis of analysis. Nevertheless, in both slices 1 and 2 of Fig. 2b, we can observe a higher concentration of magnesium, similar to horizontal grain boundaries to the analysis axis, attributed to be edges of several grains. Therefore, the four presented slices provide us the evidence of magnesium presence at both horizontal and vertical grain boundaries to the analysis axis. Additionally, the APT results provided a complementary information to High-Angle Annular Dark-Field (HAADF) images combined with Energy-dispersive X-ray spectroscopy (EDS) elemental mapping, as the location of Mg atoms was unachieved, presented in Supplementary Material Figure S1.

**Figure 3:**
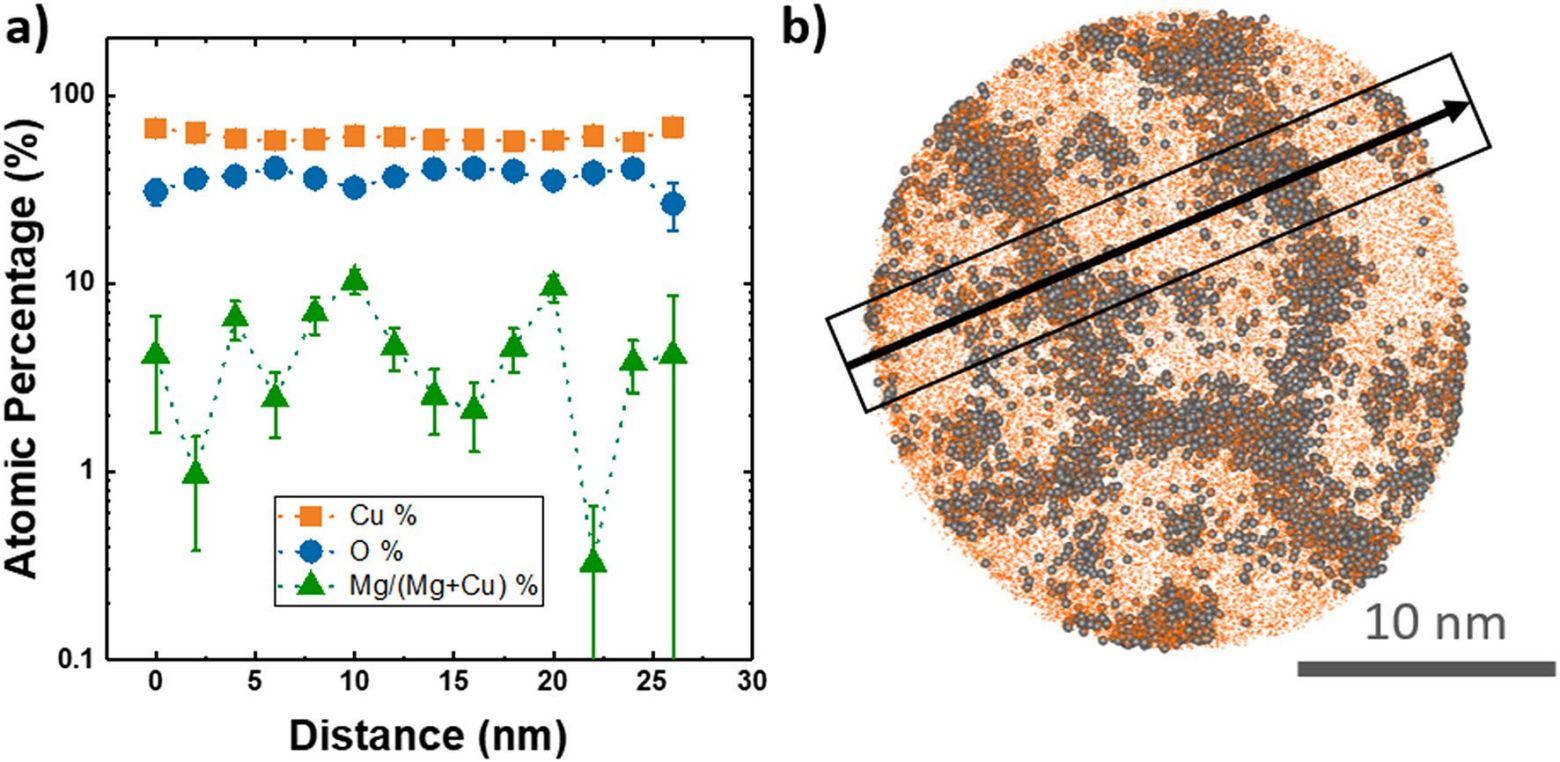
(**a**) Local atomic concentration profiles (Cu, O and Mg/(Mg + Cu) ratio) and (**b**) 2D ion map with Cu in orange and Mg in gray, extracted from a 10 nm thick slice taken across the APT specimen (plane-view of as-deposited Cu_2_O:Mg film) close to the position 4 indicated in Fig. 2a). The box and thick arrow indicate the location and direction of the profile shown in (**a**).

The quantitative analysis of the Mg distribution is hampered by the small grain size, between 10 and 20 nm, and the random orientation of the grain boundaries within the film. Hence a clear separation between the grain and a grain boundary cannot always be made in the reconstructed data. A one-dimensional concentration profile computed across the grain boundaries, identified based on the Mg^1+^ and Mg^2+^ ion distribution, Fig. 3a, reveals that the Mg/(Mg + Cu) concentration drops from ~ 7 at% to below 1 at% from the grain boundary to the bulk of the grain. A 2D ion map of Cu and Mg from a 10 nm thick slice of the APT tip is visible in Fig. 3b. However, it remains inconclusive whether the grain is fully depleted of Mg. Yet, the APT data clearly reveal the segregation of Mg atoms to the grain boundaries in the Cu_2_O film, even though the conclusion remains more qualitative at this moment. The local fluctuations in the oxygen concentration profile (Fig. 3a) should not be over interpreted, as these could originate from local electric field variations along this direction, i.e., at the grain boundaries the field is lower than in the surrounding areas. A field-dependent quantification accuracy for oxygen-containing compounds has been reported before, in which the oxygen fraction was underestimated at low field^33^.

### Temperature-dependent Hall effect measurements

The impact of the 5% Mg incorporated in the copper oxide films combined with oxidizing thermal treatments on the electrical properties was assessed via monitoring of the resistivity during different annealing steps (250, 350 and 450 °C) as well as corresponding temperature-dependent Hall effect measurements. For intrinsic Cu_2_O samples we obtained a reduction of the resistivity only during the 250 °C annealing step, leading to a final room temperature value for the resistivity of 80 ± 1 Ω cm, presented in Table 1. The annealing treatments at 350 °C and 450 °C increase the resistivity, leading to a final resistance higher than that of the original film, especially in the 450 °C case, where the resistivity reaches 1650 ± 10 Ω cm, allegedly attributed to the formation of CuO, as previously reported^17^. For the Cu_2_O:Mg thin films, we observed a different trend when compared to the intrinsic Cu_2_O samples, since all the thermal treatments lead to a reduction of the final resistivity at room temperature compared to the initial value, with the lowest resistivity value of 13.3 ± 0.1 Ω cm observed for the sample annealed at 450 °C, presented on Table 1. A more detailed discussion of the resistivity variation during each annealing treatment is included in the Supplementary Material, Figure S2.

**Table 1:** Electrical transport properties of Cu_2_O and Cu_2_O:Mg (5%) films, as-deposited and thermally annealed in air at 250 °C, 350 °C and 450 °C.

Thin films	Anneal. temp. (°C)	ρ (Ω cm)	μ (cm^2^ V^−1^ s^−1^)	p (cm^−3^)	E_A1_ (meV)	N_A1_ (cm^−3^)	E_A2_ (meV)	N_A2_ (cm^−3^)	N_A_–N_D_ (cm^−3^)
Cu_2_O	–	93 ± 1	9.7 ± 0.1	6.1 × 10^15^ ± 0.1 × 10^15^	258	7.0 × 10^17^	–	–	6.0 × 10^17^
	250	80 ± 1	8.4 ± 0.6	9.2 × 10^15^ ± 0.6 × 10^15^	249	9.5 × 10^17^	–	–	8.5 × 10^17^
	350	155 ± 2	7.3 ± 0.4	5.6 × 10^15^ ± 0.2 × 10^15^	247	5.9 × 10^17^	–	–	4.9 × 10^17^
	450	1650 ± 10	–	–	–	–	–	–	–
Cu_2_O:Mg	–	61.3 ± 0.4	2.4 ± 0.1	4.3 × 10^16^ ± 0.2 × 10^16^	251	4.0 × 10^18^	124	4.7 × 10^16^	4.0 × 10^18^
	250	24.4 ± 0.2	2.8 ± 0.2	9.3 × 10^16^ ± 0.5 × 10^16^	251	7.7 × 10^18^	113	1.2 × 10^17^	7.7 × 10^18^
	350	27.7 ± 0.1	2.7 ± 0.5	8.8 × 10^16^ ± 0.5 × 10^16^	251	6.3 × 10^18^	126	1.4 × 10^17^	6.3 × 10^18^
	450	13.3 ± 0.1	2.3 ± 1	2.9 × 10^17^ ± 1.8 × 10^17^	251	9.5 × 10^19^	136	2.2 × 10^17^	9.5 × 10^19^

Resistivity, mobility and charge carrier density values were determined at room temperature by Hall effect measurements. Ionization energies and concentrations of the two acceptor levels, as well as the difference between acceptor and donor concentrations are obtained from the fitting of temperature-dependent Hall carrier density.

The electrical nature of the defects formed during the thermal treatments was then evaluated by temperature-depend Hall effect measurements from 220 to 400 K. The results thereby confirmed that all samples indeed showed p-type conductivity. Table 1 shows the effect of the annealing temperature on mobility and charge-carrier density of the intrinsic and Mg-doped Cu_2_O films. For the intrinsic Cu_2_O case, the as-deposited sample and the samples annealed below 450 °C show a mobility maintained roughly constant at about 7–9 cm^2^ V^−1^ s^−1^, consistent with a grain-boundary-limited type of conduction in randomly oriented Cu_2_O grains^34–36^. The sample annealed at 450 °C, reached a resistivity above 1000 Ω cm, exceeding the experimental range of the setup, as CuO is formed in particularly at the grain boundaries, which leads to a decrease of mobility, as previously reported^29^. The free charge carrier density, associated to holes, varied in the Cu_2_O samples showing a maximum Hall carrier density of 9.2 × 10^15^ cm^−3^ for the sample annealed at 250 °C. In the case of the Cu_2_O:Mg films, the carrier mobility ranges between 2 and 3 cm^2^ V^−1^ s^−1^, i.e. lower than for intrinsic sample, while the charge carrier density increases after thermal annealing treatments, presenting a maximum for the film annealed at 450 °C, with a value of 2.9 × 10^17^ cm^−3^.

**Figure 4:**
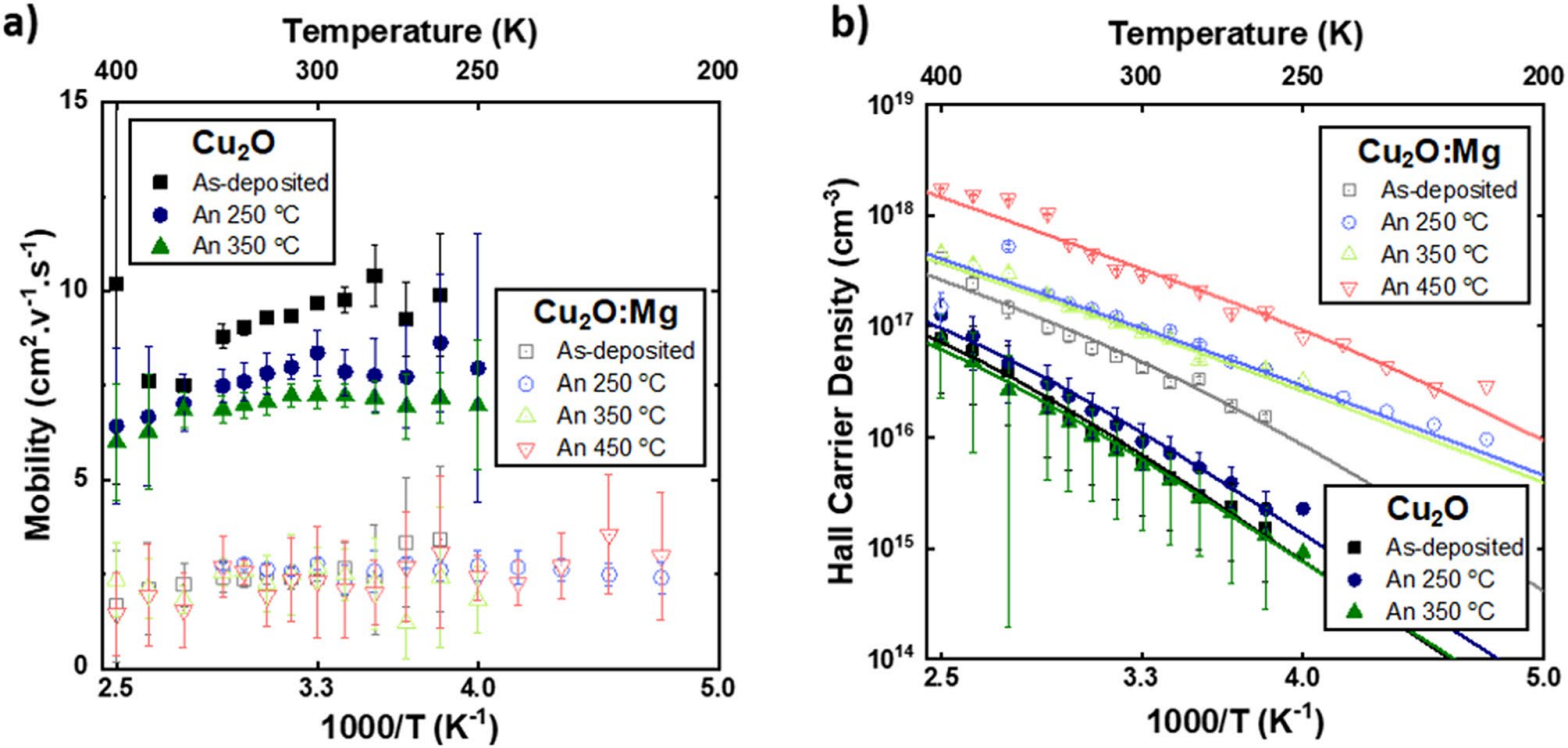
Temperature-dependent Hall effect measurements of Cu_2_O and Cu_2_O:Mg thin films. (**a**) Mobility (μ) and (**b**) Hall carrier density (p) as a function of the reciprocal temperature, where the lines correspond to the charge balance equation fit.

Figure 4 shows the temperature dependence of the mobility and carrier density obtained from Hall effect measurements in the range 220–400 K for the two groups of films, Cu_2_O and Cu_2_O:Mg. The data obtained for the resistivity are given in Supplementary Material, Figure S3. The curves of the carrier mobility in intrinsic films show small variations in the temperature range of the experiment, while constant values are obtained for the Cu_2_O:Mg films. The Cu_2_O film in the as-deposited state has the highest carrier mobility values, reducing with increasing annealing temperature. No clear trend is observed for the doped films. The Hall carrier density is reduced with temperature decrease, as expected for an oxide semiconductor, with the undoped Cu_2_O group presenting lower values.

The curves obtained for the temperature-dependent Hall carrier densities for the Cu_2_O and Cu_2_O:Mg thin films (Fig. 4b) have been fitted using a two acceptor levels model. Similar to previous studies^37^, the numerical solution p(T) of the charge balance equation (CBE) allows us to obtain the influence of temperature on the Cu_2_O carrier concentration, while having in consideration Cu_2_O as a nondegenerate p-type semiconductor:

$$p\left( T \right) + N_{D} = \frac{{N_{A1} }}{{1 + p\left( T \right).\frac{{g_{A1} }}{{N_{V} \left( T \right)}} e^{{\frac{{E_{A1} }}{{k_{b} T}}}} }} + \frac{{N_{A2} }}{{1 + p\left( T \right).\frac{{g_{A2} }}{{N_{V} \left( T \right)}} e^{{\frac{{E_{A2} }}{{k_{b} T}}}} }}$$

$$N_{V} \left( T \right) = 2\left( {2\pi m_{h}^{*} k_{B} T} \right)^{\frac{3}{2}} /h^{3}$$

where $N_{A1}$, $N_{A2}$ and $N_{D}$ are atomic densities for, respectively, the first and second acceptor, and the donor, $E_{A1}$ and $E_{A2}$ are the acceptor ionization energies and $N_{V}$ is the effective density of states in the valence band. The hole effective mass $m_{h}^{*}$ was fixed as 0.58 m_0_ as proposed by Hodby et al*.*^38^, $h$ and $k_{B}$ are Planck’s and Boltzmann’s constants, respectively. As the degeneracy factors are the ratios between the occupied-state and unoccupied-state degeneracies and as the negative spin − orbit splitting suggests to use an unoccupied-state degeneracy of 2 and an occupied state degeneracy of 1, we used the same degeneracy factor for $g_{A1}$ and $g_{A2}$ of 1/2, as proposed by Brochen et al*.*^13^. In order to allow a simplified fitting procedure using the CBE, the donor density ($N_{D}$) was fixed at 3 × 10^17^ cm^−3^ in all the different sets of samples. The values of the different acceptor levels concentrations, ionization energies and the difference between acceptors and donor density for each Cu_2_O and Cu_2_O:Mg case are given in Table 1.

In the case of the intrinsic Cu_2_O for the films in the as-deposited state and annealed at 250 °C and 350 °C, only one deep acceptor level is found with an acceptor ionization energy ($E_{A1}$) of 251 ± 7 meV, which is generally attributed to the energy of simple copper vacancies, $V_{Cu}^{^{\prime}}$, in theoretical^39^ and experimental^40^ studies. Concerning the concentration of this acceptor state ($N_{A1}$), the highest value of 9.50 × 10^17^ cm^−3^ was found for the sample annealed at 250 °C, which presents the lowest resistivity value of the intrinsic Cu_2_O samples.

In the Mg-doped cases, the introduction of a second acceptor level led to an improvement of the model. In order to reduce the number of fitting parameters, it was decided to maintain the ionization energy of the first acceptor level constant, fixed at 251 meV, corresponding to the average value obtained for the undoped Cu_2_O samples. Regarding this first acceptor level, its concentration ($N_{A1}$) increases up to 9.5 × 10^19^ cm^−3^ for the Cu_2_O:Mg 450 °C annealed sample. This is attributed to an increase of copper simple vacancies ($V_{Cu}^{^{\prime}}$) which is the main mechanism for the reduction of resistivity^41,42^ induced by the presence of the magnesium. For the second acceptor level ($E_{A2}$), we found an ionization energy of 125 ± 9 meV with an increasing acceptor concentration ($N_{A2}$) with the annealing temperature, reaching 2.2 × 10^17^ cm^−3^ for the 450 °C annealing temperature.

## Discussion

The incorporation of magnesium in copper oxide leads to changes on the grain growth of the thin films. The grains, with a size between 10 and 20 nm, are characterized by a round-like shape, contrarily to the columnar growth previously reported^29,43^. The APT measurements confirm the presence of the magnesium atoms with a concentration of 5 at% throughout the whole film. However, the dopant is mostly localized at the grain boundaries of the copper oxide polycrystalline film, with the concentration of 7 at% at this region.

The segregation of the magnesium ion has direct impacts on the electronic transport in the oxide semiconductor. We observe a reduction of Hall carrier mobility, which can be attributed to an increase of hole scattering, related to the magnesium incorporation. Copper oxide usually presents a grain-boundary-limited type of conduction^34^ that governs the electronic transport in the material. Due to the presence of the dopant at the grain boundaries and its effect on the grain growth, we assume that an increase of hole scattering appears at the boundaries of Cu_2_O:Mg grains. Therefore, we assume that the presence of magnesium will directly influence the grain-boundary mobility, causing the reduction to the 2.3–2.8 cm^2^.V^−1^.s^−1^ range in the Cu_2_O:Mg thin films. Additionally, the presence of magnesium inside the grain could also reduce the bulk mobility. Nevertheless, this contribution will not affect the total mobility, as it is a less significant mobility term. Moreover, the temperature-dependent mobility values are roughly constant with the increase of temperature in both sets of samples, which is consistent with a grain-boundary-limited type of conduction in randomly-oriented Cu_2_O grains.

In our modelling approach, we considered the thin film conductivity, mobility and carrier density without looking at the spatial distribution of the Mg dopants, since the Hall effect technique is applied on the whole sample and thus sensitive to both bulk and grain boundaries. In general, assuming Matthiessen's rule, the mobility is affected by several scattering mechanisms and the smaller one is responsible for the limitation of the overall mobility value. In the case of Cu_2_O:Mg film, it is difficult to observe any clear temperature dependence for the mobility, and its value is well reduced compared to undoped Cu_2_O films. For these layers, considering that the Mg distributions obtained from APT technique show higher Mg density in the grain boundaries, one could expect a higher value for the trap density at the grain boundary compared to undoped Cu_2_O films, which could affect carrier mobility. In Nguyen et al.^44^, we can find a clear correlation between trap density at the grain boundaries and free carrier density in highly-doped polycrystalline conductive oxides. We find higher carrier concentrations for Cu_2_O:Mg films compared to undoped Cu_2_O films, which could be related to trap density increase at grain boundaries. Our approach does not consider GB-limited scattering effects, thus we cannot exclude that the second impurity level could be related to the Mg effective doping inside grains and/or a possible influence of the high Mg density at grain boundaries, as observed by APT.

Although the magnesium concentration inside the grain is below 1%, its mere presence induces the formation of a shallower acceptor value with an ionization energy of 125 mV, comparable to the secondary defect observed in Cu_2_O:Sr^13^. This type of defect is attributed to a large size impurity−vacancy complex found previously in Sr-doped Cu_2_O, as this defect is a clustering of a two-simple vacancy with the dopant in a tetrahedral position, $\left[ {Mg_{i} - 2V_{Cu}^{^{\prime}} } \right]^{ - }$, previously proposed by Isseroff and Carter^42^. A representation of the suggested $\left[ {Mg_{i} - 2V_{Cu}^{^{\prime}} } \right]^{ - }$ complex is shown in the Supplementary Material Figure S4. It is expected that this complex prevents the formation of split vacancies for a single cation vacancy, since the divalent cation would assume a position similar to a split copper vacancy (*V*_*split*_) in the crystallographic structure^42^. As a consequence, Cu_2_O:Mg materials present an increased response in photoconductivity^14^ and exhibit improved chemical stability at high temperatures^14,17^, as already reported in the literature. Additionally, this complex would further increase the charge carrier density after annealing treatments in oxidizing conditions, by a simple copper vacancy doping mechanism assisted by the magnesium incorporation^13^.

The incorporation of different dopants in copper oxide has various types of effects on the electronic transport in the material.

In Table 2, we compare the resistivity, mobility and acceptor level energy values of several Cu_2_O doping systems reported in literature. When the resistivity values are reduced by the presence of the dopant, an extra acceptor level with a reduced value or range of activation energy is observed, when compared to the energy level corresponding to copper simple vacancies ($V_{Cu}^{^{\prime}}$), around 210 meV. The $V_{Cu}^{^{\prime}}$ defects are known to increase the number of free charge carriers and, consequently, they act as dopant species by reducing the resistivity. Furthermore, the generation of free charge carriers is also impacted by the acceptor complex associated with the extrinsic atom. For instance, doping with nitrogen reduces the resistivity down to 1.1 Ω cm, and increases the hole density up to 10^17^ cm^−3^, by creating an extra acceptor level in Cu_2_O at 140 meV^9^. In silicon-doped and strontium-doped films, the charge carriers’ density can be further increased to 10^17^ cm^−3^, which reduces the resistivity to the 1–10 Ω cm range. In these cases an energy value of a large size impurity−vacancy complex was found at 133 and 190 meV, for strontium^13^ and silicon^45^, respectively. In a simple, more straightforward analysis, the presence of dopants such as Mg, Sr or Si in Cu_2_O structures would induce a donor-type doping, due to their cation behaviour. Therefore, the resulting p-type conductivity of such extrinsically-doped Cu_2_O films is attributed to a charge compensation mechanism, based on the production of simple copper vacancies that leads to an increase of charge carriers, as suggested by Isseroff and Carter^42^ and Minami et al*.*^12,46^.

**Table 2:** Electrical properties of dopants used in Cu_2_O thin films: resistivity, carrier concentration at room temperature and acceptor level energy.

Dopant	Amount of dopant (%)	Resistivity (Ω cm)	Carrier concentration (cm^−3^)	Acceptor level (eV)	References
Pure Cu_2_O	0	150	1.5 × 10^15^	210	^47^
N^−^	< 0.1	15.2	7 × 10^16^	121–140	^9,11^
F^−^	0.9	11.6	1.9 × 10^16^	–	^48^
Cl^−^	–	200	8.6 × 10^13^	330	^49^
Na^+^	–	0.5	2 × 10^19^	22–46	^12,46^
Zn^2+^	8.2	100	7 × 10^16^	470	^50^
Ni^2+^	2	100	2 × 10^15^	220–230	^51^
Sr^2+^	5	1.2	2.8 × 10^17^	104–149	^13^
Nd^3+^	5	85	21.5 × 10^15^	–	^52^
Si^4+^	0.58	12	1 × 10^17^	190	^45^
Mg^2+^	5	13.3	2.9 × 10^17^	113–136	This work

In conclusion, magnesium-doped copper oxide thin films present improved electrical properties when compared to intrinsic thin film, especially after thermal treatments. The dopant is found mostly at the grain boundary of the Cu_2_O, as confirmed by Atom Probe Tomography, which reaches 5% in atomic percentage. The magnesium segregation hinders the mobility of these films, reducing it to 2–3 cm^2^/V s. However, the small quantity, below 1%, that is present inside the grain is responsible for the drastic increase of the charge carrier density up to 2.9 × 10^17^ cm^−3^, for the film annealed at 450 °C. Temperature-dependent Hall effect measurements led to the determination of a first acceptor level in the range of 247–258 meV attributed to the copper simple vacancy. An additional acceptor level with an activation energy around 113–136 meV was found for the magnesium-doped samples. The use of annealing treatments coupled with the Mg presence in Cu_2_O thin films provides new insights into the dopant influence for the copper vacancy generation mechanism.

## Methods

### Films deposition

A butanol-based solution containing the precursors was used for the deposition by Aerosol-Assisted Metal Organic Chemical Vapour Deposition (AA-MOCVD). The total concentration of the solution was fixed at 30 mM in all cases. The first solution was composed of pure copper acetylacetonate, Cu(acac)_2_ [Sigma Aldrich], while in the second one magnesium acetylacetonate, Mg(acac)_2_ [Sigma Aldrich] was added to the solution. The Mg/(Mg + Cu) ratio in the later solution was fixed at 33 at%, as this level was known to produce sufficiently low resistivity materials in our previous study^16^. Ethylendiamine [C_2_H_8_N_2_, Sigma Aldrich] with a concentration of 40 mM was added to increase the chemical precursor solubility. The temperature of the substrates, Corning glass 1737 and p*-*type Si(100), was fixed at 350 °C. The deposition time was 3 h and the solution consumption rate was 1.5 ml min^−1^ with an argon flow of 6 l min^−1^ and oxygen flow of 2.5 l min^−1^, resulting in an O_2_ presence of 29%.

The thermal annealing treatments were performed on a hot-plate exposed to air, coupled with an *in-situ* resistance measurement setup based on a 2-probe system including a KEITHLEY 2400 source-meter. Electrodes on both sides of the samples were made with silver paste. Three stages of temperature (T_a_) were chosen, 250 °C, 350 °C and 450 °C, all for 30 min, while the heating and cooling rate was kept constant at 10°C/min.

### Characterization techniques

Scanning Electron Microscopy (SEM) and Energy-dispersive X-ray spectroscopy (EDS) were conducted in a FEI Quanta 250 FE-ESEM tool, with an energy beam of 5000 eV. Images of the film cross section were used to measure the film thickness. Transmission electron microscopy (TEM) imaging was obtained with a JEOL JEM 2010 microscope operating at 200 kV (0.19 nm resolution), provided with an EDS system, INCA Energy TEM 100 X-Max 65 T. Cross-sectional samples were prepared in Cu_2_O:Mg film on silicon by tripod polishing resulting in a sample thickness of about 10 μm, glued to a copper grid. Argon ion beam milling was used until perforation of the interface.

Atom probe specimens were prepared using the standard focused ion beam (FIB) lift-out technique (e.g. Thompson et al*.*^53^ and Miller et al.^54^) in a dual-beam FIB-SEM setup operating with a Ga^+^ ion source (G3CX from Thermofisher). Laser-assisted atom probe analysis was performed in a reflectron-fitted LEAP 5000XR, equipped with an UV laser (355 nm). The specimen, the as-deposited Cu_2_O:Mg film, was analysed at 50 K base temperature, with 25 pJ laser pulses applied at a repetition rate of 125 kHz. In total ~ 2.400.000 ions were collected, which corresponds to the full Cu_2_O film. The analysis was stopped manually in the Si substrate, a few nm after the Cu_2_O/Si interface. 3D data reconstruction was performed in IVAS 3.8 using the tip profile evolution taken from an SEM image before APT analysis (Fig. 2d). The image compression factor (ICF), the field factor (k) and the evaporation field (F) were set to 1, 3.3 and 30 V/nm (Cu) so as to achieve a flat Si iso-concentration (1 at%) surface at the Cu_2_O/Si interface and a Cu_2_O film thickness of around 200 nm, as was measured by SEM from the initial APT specimen. Further details about the APT technique are included in Supplementary Material.

The temperature-dependent transport properties were obtained using a Van der Pauw configuration in a HMS5500 Hall effect measurement setup from Ecopia Microworld. Circular Ag ohmic contacts of 500 µm diameter were deposited at the four corners of 5 × 5 mm^2^ size samples deposited on Corning Glass by electron beam evaporation. The electrical properties were carried out in the temperature range of 80–350 K using liquid Nitrogen cooling, with a permanent magnetic field of 0.57 T. Mobility and carrier concentration values as well as the corresponding error bars were calculated from the average and standard deviation of five different measurements per temperature.

## Supplementary Information

Supplementary Information
