# Author Correction: Grain-boundary segregation of magnesium in doped cuprous oxide and impact on electrical transport properties

**DOI:** 10.1038/s41598-021-96650-8

**Published:** 2021-08-25

**Authors:** João Resende, Van-Son Nguyen, Claudia Fleischmann, Lorenzo Bottiglieri, Stéphane Brochen, Wilfried Vandervorst, Wilfried Favre, Carmen Jiménez, Jean-Luc Deschanvres, Ngoc Duy Nguyen

**Affiliations:** 1 CNRS, Grenoble INP, LMGP, Univ. Grenoble Alpes, 38000 Grenoble, France; 2 Département de Physique, CESAM/Q-MAT, SPIN, Université de Liège, 4000 Liège, Belgium; 3 CEA, INES, LITEN, 50 Avenue du lac Léman, 73375 Le Bourget-du-lac, France; 4 IMEC, Kapeldreef 75, 3001 Heverlee, Belgium; 5 Instituut Voor Kern- en Stralingsfysica, KU Leuven, Celestijnenlaan 200D, 3001 Leuven, Belgium

Correction to: *Scientific Reports* 10.1038/s41598-021-86969-7, published online 08 April 2021

The Acknowledgements section in the original version of this Article was incomplete.

“Financial support by the IDS-FunMat scholarship selected under the program “ERASMUS MUNDUS II 2009-2013” is gratefully acknowledged. N. D. N. acknowledges the financial support by the F.R.S-FNRS of Belgium (project J.0124.19) and by the European Joint Doctorate FUNMAT (H2020-MSCA-ITN-2014, Project ID 641640). This project was financially supported by ‘‘Carnot Energies du Futur’’ (SOLAROX project). This work benefited from the facilities and expertise of the OPE)N(RA characterization platform of FMNT (FR 2542, fmnt.fr) supported by CNRS, Grenoble INP and UGA. The authors would like to thank to Laetitia Rapenne for the TEM analysis.”

now reads:

“Financial support by the IDS-FunMat scholarship selected under the program “ERASMUS MUNDUS II 2009-2013” is gratefully acknowledged. N. D. N. acknowledges the financial support by the F.R.S-FNRS of Belgium (project J.0124.19) and by the European Joint Doctorate FUNMAT (H2020-MSCA-ITN-2014, Project ID 641640). This project was financially supported by ‘‘Carnot Energies du Futur’’ (SOLAROX project). This work benefited from the facilities and expertise of the OPE)N(RA characterization platform of FMNT (FR 2542, fmnt.fr) supported by CNRS, Grenoble INP and UGA. The authors would like to thank to Laetitia Rapenne for the TEM analysis. C. F. acknowledges the financial support by FWO-Hercules through projects ZW13_09 and AKUL/15/22.”

Additionally, in the original version of this Article, João Resende was incorrectly listed as the corresponding author. The correct corresponding author for this Article is Ngoc Duy Nguyen. Correspondence and request for materials should be addressed to ngocduy.nguyen@uliege.be.

The original Article has been corrected.

