# Supplementary material for: Grain-boundary segregation of magnesium in doped cuprous oxide and impact on electrical transport properties
Source: Sci Rep. 2021 Apr 8;11:7788. doi: 10.1038/s41598-021-86969-7 (PMC8032787; doi:10.1038/s41598-021-86969-7)
Supplement: Supplementary file 1 — Supplementary Information [file 41598_2021_86969_MOESM1_ESM.docx]

# Grain-Boundary Segregation of Magnesium in Doped Cuprous Oxide and Impact on Electrical Transport Properties

**João Resende^*,1, 2^, Van-Son Nguyen­^3^, Claudia Fleischmann^4^, Lorenzo Bottiglieri^1^, Stéphane Brochen^1^, Wilfried Wandervorst^4,5^, Wilfried Favre^3^, Carmen Jiménez^1^, Jean-Luc Deschanvres^1^ and Ngoc Duy Nguyen^2^**

^1^ Univ. Grenoble Alpes, CNRS, Grenoble INP, LMGP, F-38000 Grenoble, France

^2^ Département de Physique, CESAM/Q-MAT, SPIN, Université de Liège, B-4000 Liège, Belgium

^3^CEA, INES, LITEN, 50 Avenue du lac Léman, 73375 le Bourget-du-lac, France

^4^ IMEC, Kapeldreef 75, 3001 Heverlee, Belgium

^5^ Instituut voor Kern- en Stralingsfysica, KU Leuven, Celestijnenlaan 200D, 3001 Leuven, Belgium

# Supplementary Information

**Atom Probe Tomography (APT) experimental details**

Laser-assisted APT is based on the controlled field ionization and evaporation of surface atoms (one-by-one) from a needle-shaped tip, under the combined effect of a high electric field and a short laser pulse. The evaporated ions are subsequently accelerated by the electric field towards a detection system to record their flight time (chemical analysis), impact position, evaporation sequence and number of counts. The spatial distribution of the elements on the detector is a magnified (10^6^X) view of their distribution on the specimen apex whereby the evaporation sequence can be converted into a depth scale. Combining all these data, a complete elemental distribution in 3D can be reconstructed with sub-nm resolution (Figure 2a) in the main article text). Prior to specimen preparation, the sample was capped ex-situ with a 50 nm Ni layer deposited by thermal evaporation. The region of interest was protected against ion beam damage with a 300 nm Pt layer, using ion-beam assisted deposition in the FIB-SEM setup. The standard lift-out and annular milling protocol was followed with a 30 kV Ga^+^ beam, including a final cleaning step with a 5 kV ion beam. The Atom probe tomography specimens were mounted on a commercially available microtip coupon from Cameca, which was loaded immediately into the atom probe chamber for analysis.

**High-Angle Annular Dark-Field (HAADF) and Energy-Dispersive X-Ray Spectroscopy (EDS)**

The HAAD images presented in Figure S1 show the Mg presence throughout the film. Nevertheless, the spatial resolution of the EDS elemental maps is not sufficient to find the location of the Mg.


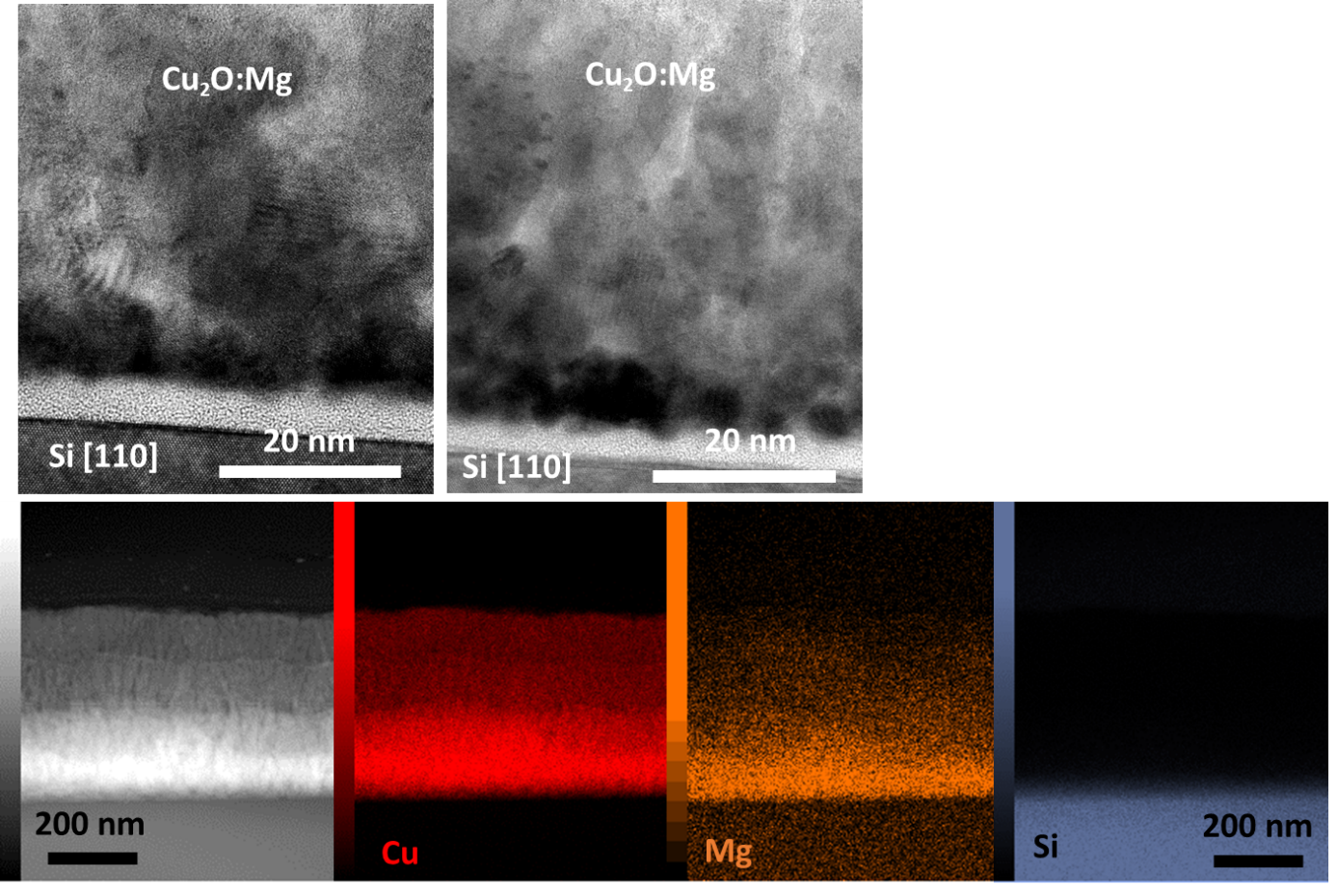


Figure S1 HAADF of as-deposited Cu_2_O:Mg with the EDS maps of Cu, Mg and Si.

**Resistivity measurements during annealing treatments and Hall effect measurements of Cu_2_O and Cu_2_O:Mg thin films**

The electrical properties impact of the magnesium incorporation in copper oxide combined with oxidizing thermal treatments was accessed via monitoring of the resistivity *in-situ* during different annealing steps. In these experiments, the resistance was measured with a 2-probe system while the sample was placed on a hot plate. This setup allowed us to observe the resistance variation during the heating and the cooling stages, as well as the effect during the annealing step at a given temperature. To circumvent the difference in sample thickness and distance of the electrodes, we converted the resistance data into resistivity values accordingly the Van der Pauw measurements performed before the experiment at room temperature, presented in Table 1, in the main article text. The temperature dependence of the resistivity, dressed in Arrhenius representations for the intrinsic and Mg-doped Cu_2_O films, are shown in Figure S2 a), b) and c) for the three annealing temperatures. In all cases, we have a reduction of the resistivity with the increase of the temperature, as expected in non-degenarated semiconductors. In the undoped Cu_2_O case, after the annealing treatments, we observed a reduction of resistivity for the 250 °C case, while the other treatments at higher temperatures lead to an increase of overall resistivity values. This is expected due to the formation of CuO in the thin films, as previously reported.^1,2^ Nevertheless, in the Cu_2_O:Mg thin films, all the thermal treatments lead to a reduction of resistivity, with the lowest value of 13.3 ± 0.1 Ω.cm observed for the sample annealed at 450 °C.

Figure S2 Dependence of the resistivity of the Cu_2_O thin films with inverse of the temperature during the 3 different annealing treatments: a) 250°C, b) 350°C and c) 450°C. Grey and red triangles visible in b) to help the visualization of different slopes.

In a general analysis of all the annealing treatments, the influence of the temperature, represented by the slope of the curves, is larger for the intrinsic Cu_2_O films than for the Cu_2_O:Mg films, during both heating and cooling stage. Based on the variation of the resistivity during the heating and cooling down stages of the annealing treatment, it is possible to extract the activation energy in both Cu_2_O types of film. Similar to previous studies on the influence of temperature on the Cu_2_O resistivity^3^, the linearization of these curves allows us to analyse through the use of an Arrhenius equation, required for the extraction of the activation energies, namely, $\rho=\rho_{0}e^{\frac{E_{a}}{k_{b}T}},$where *ρ* is the resistivity, *ρ_0_* a constant, *E_a_* is the activation energy and *k_b_* is the Boltzmann constant.

In the case of the intrinsic Cu_2_O samples and for the films annealed at 250°C and 350°C the curves are linear and the slopes during heating and cooling are similar. In this intrinsic case the average activation energy is of 237±20 meV. which are normally attributed to the formation energy of simple cooper vacancies, $V_{Cu}^{'}$, in theoretical ^4^ and experimental ^5^ studies. However, for the annealing at 450°C, the slope is higher in the cooling step, especially at high temperatures (Figure S2c)). We relate this phenomenon to the higher quantity of CuO, formed at high temperature, which changes the resistivity behaviour under temperature variation in a non-reversible way.

The samples containing magnesium present a different characteristic than those of the intrinsic Cu_2_O, in particular on the heating part of the experiment. There is a clear change of slope at around 170°C - associated to a reciprocal temperature of 2.25 (1000/K) - for all 3 annealing curves. By taking this aspect into consideration, we separate the heating ramp of the annealing into different sections, one from room temperature to 170°C, and another one from 170°C until the designated annealing temperature. Considering the low temperature regime, the activation energy varies between 140mV and 166mV, showing an average activation energy of 157 ±20 meV. At a higher temperature, the activation energy increases with the temperature, from 209 meV to 250 meV, with energies comparable to the heating activation energy for the intrinsic case, around 237 meV. This evidence of two different energies observed for the Cu_2_O:Mg thin films, lead us to use a two-level acceptor model to fit the Hall effect data presented in the main article text.

In Figure S3, we observe the temperature dependence of the resistivity values during the Hall effect measurements, for the intrinsic Cu_2_O and Cu_2_O:Mg films, as-deposited and after the different annealing treatments: 250°C, 350°C and 450°C. The difference of slope between the Cu_2_O and Cu_2_O:Mg groups are once again apparent, with the intrinsic cases showing a higher slope. The fit of the resistivity was based on the numerical solution of the charge balance equation (CBE) and the mobility values from the Hall measurements, represented by the coloured lines in Figure S3, showing a good agreement with the experimental data and carriers model. In the Cu_2_O:Mg sample annealed at 450 °C, we observe a significant difference between the experimental data and the model, caused by the large variation on mobility for this sample, between 1 and 2.5 cm^2^.V^-1^.s^-1^.


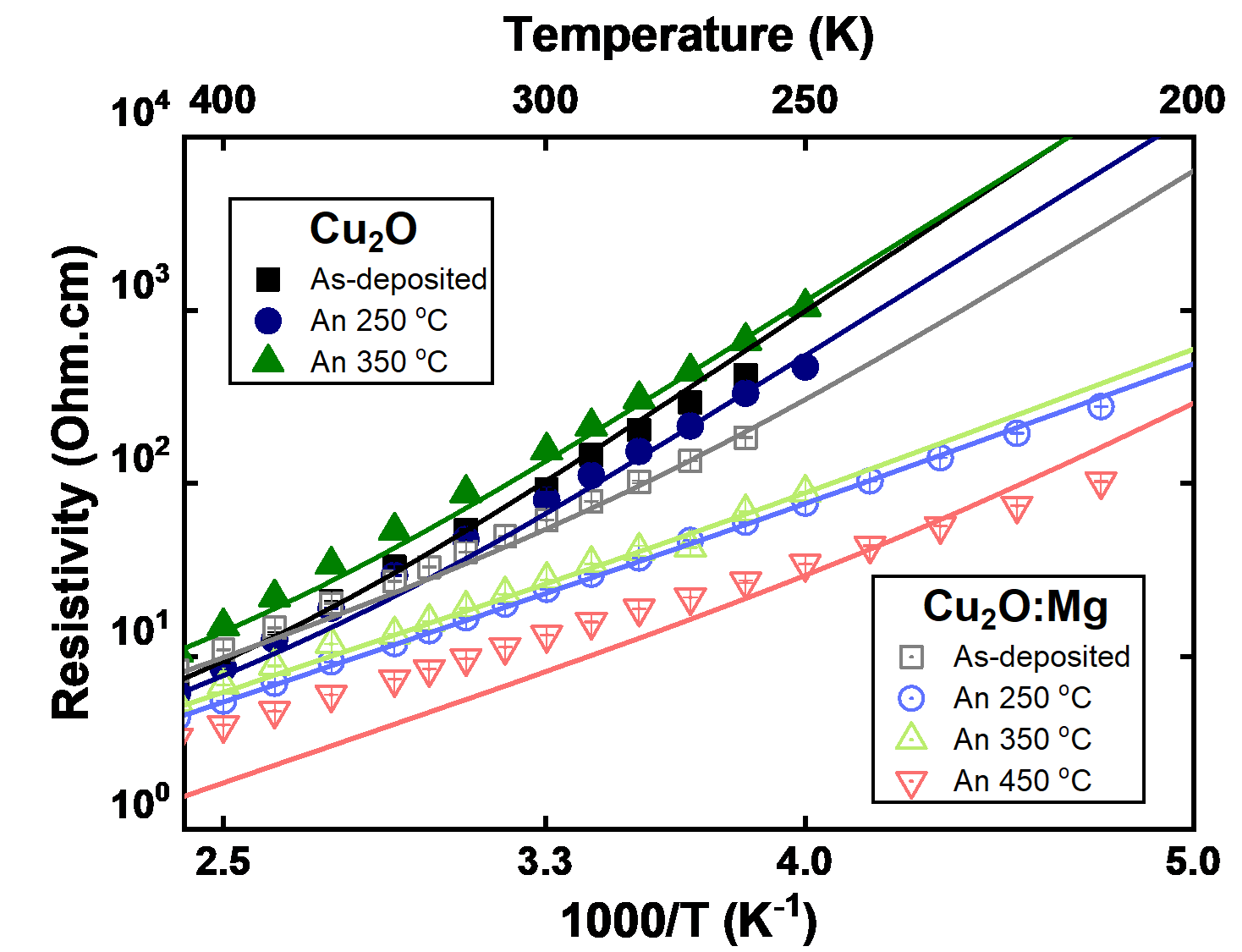


Figure S3 Temperature dependent resistivity values based on Hall effect measurements of Cu­_2_O and Cu_2_O:Mg thin films, as a function of the reciprocal temperature, where the plotted lines correspond to model used to fit the data.

In Figure S4, we represent a clustering of a two-simple vacancy with the dopant in a tetrahedral position, $\left[ Mg_{i}-2V_{Cu}^{'} \right]^{-}$,which has been proposed by Isseroff and Carter.^6^ This defect can explain the ionization energy of 125 ± 9 meV found for the secondary acceptor.


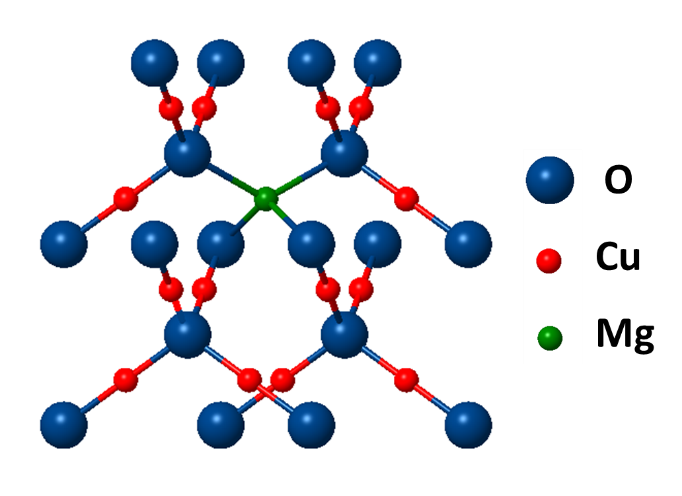


Figure S4 Proposed magnesium complex in a tetrahedral site, assisted by two copper vacancies. Copper atoms pictured as red circles, oxygen as blue ones and magnesium as green ones. Representation prepared by VESTA software^7^

## References

1. Resende, J. *et al.* Resilience of Cuprous Oxide under Oxidizing Thermal Treatments via Magnesium Doping. *J. Phys. Chem. C* **123**, 8663–8670 (2019).

2. Sohn, J. *et al.* Effects of vacuum annealing on the optical and electrical properties of p-type copper-oxide thin-film transistors. *Semicond. Sci. Technol.* **28**, 015005 (2013).

3. Tapiero, M., Zielinger, J. P. & Noguet, C. Electrical conductivity and thermal activation energies in Cu_2_O single crystals. *Phys. Status Solidi* **12**, 517–520 (1972).

4. Nolan, M. & Elliott, S. D. Tuning the transparency of Cu_2_O with substitutional cation doping. *Chem. Mater.* **20**, 5522–5531 (2008).

5. Figueiredo, V. *et al.* p-Type CuO Thin-Film Transistors Produced by Thermal Oxidation. *J. Disp. Technol.* **9**, 735–740 (2013).

6. Isseroff, L. Y. & Carter, E. A. Electronic structure of pure and doped cuprous oxide with copper vacancies: Suppression of trap states. *Chem. Mater.* **25**, 253–265 (2013).

7. Momma, K. & Izumi, F. VESTA 3 for three-dimensional visualization of crystal, volumetric and morphology data. *J. Appl. Crystallogr.* **44**, 1272–1276 (2011).
